# Plasmacytoid Dendritic Cells Exhibit High Transferrin Receptor Expression Without Iron Accumulation

**DOI:** 10.1002/eji.70219

**Published:** 2026-06-17

**Authors:** Carrie Corkish, Simon O'Shaughnessy, Claire Lin, Sophia Wugk, Sarah Kenny, Linda Sinclair, Suzanne Cloonan, David Finlay

**Affiliations:** ^1^ School of Biochemistry and Immunology Trinity Biomedical Sciences Trinity College Dublin Dundee Ireland; ^2^ School of Life Sciences University of Dundee Dundee UK; ^3^ School of Medicine Trinity Biomedical Sciences Trinity College Dublin Dundee Ireland; ^4^ School of Pharmacy and Pharmaceutical Sciences Trinity Biomedical Sciences Trinity College Dublin Dundee Ireland

**Keywords:** CD71, dendritic cells, interferons, metabolism, nutrient transport, pDC, plasmacytoid, proteomics, TFRC, transferrin receptor

## Abstract

Plasmacytoid dendritic cells (pDCs) are specialized antiviral sentinels defined by rapid type I interferon (IFN‑I) production, yet their proteomic organization and metabolic requirements remain incompletely understood. We established the steady‑state proteome of murine splenic pDCs directly ex vivo using deep, absolute quantitative mass spectrometry and compared it with conventional dendritic cell subsets and human pDCs. pDCs exhibited a highly conserved proteomic architecture across species, with selective divergence in central carbon metabolism, amino‑acid utilization, and nutrient transporter expression. Notably, pDCs expressed exceptionally high levels of the transferrin receptor (TFRC) in both mice and humans and displayed robust transferrin‑mediated iron uptake relative to other splenic immune populations. Despite this, pDCs did not demonstrate increased total cellular iron or enhanced ferritin‑based storage. Instead, proteome‑wide iron mapping revealed preferential allocation of iron to functional iron‐sulfur and heme‑containing proteins, particularly within mitochondrial pathways. Detection of the iron exporter ferroportin indicated coordinated iron import and efflux, establishing sustained iron flux rather than net accumulation. Functional assays showed that iron availability does not constrain TLR9‑induced IFN‑I or TNF production. Together, these data define a conserved iron‑handling program in pDCs characterized by high TFRC expression, balanced iron flux, and targeted redistribution into essential protein systems.

## Graphical abstract

• pDCs are defined by high surface TFRC expression and dynamic iron flux, distinguishing them from conventional dendritic cell subsets.

• In the Iron proteome, pDC are enriched for mitochondrial heme and Fe–S proteins but have similar iron storage to cDC.

• Iron chelation does not disrupt pDC type‐1 IFN production.

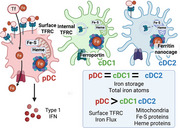

## 1 Introduction

Plasmacytoid dendritic cells (pDCs) are a distinct subset of dendritic cells specialized in the detection of viral nucleic acids and are found in lymphoid tissues and peripheral blood in both humans and mice [1]. pDCs are professional producers of type I interferons (IFN‑α/β), secreting large quantities of IFN‑α in response to viral ligands sensed primarily through endosomal Toll‑like receptors TLR7 and TLR9. Through this rapid interferon response, pDCs play a central role in antiviral immunity and in shaping downstream adaptive immune responses [1]. In the steady state, pDCs differ markedly from conventional dendritic cells (cDCs), exhibiting a rounded, lymphoid‑like morphology, low basal expression of MHC class II and co‑stimulatory molecules, and limited capacity to prime naïve T cells. Upon activation, pDCs undergo morphological and transcriptional remodeling, upregulating antigen‑presentation and co‑stimulatory pathways, although their T‑cell priming capacity remains lower than that of cDCs [2]. Unlike proliferating lymphocytes, pDCs are terminally differentiated, nondividing cells in the steady state.

While pDC identity and function have been extensively characterized using transcriptional and cytometric approaches, quantitative proteomic analyses of pDCs, particularly across species, remain limited. This represents a critical gap, as protein abundance more directly reflects the molecular machinery governing cellular metabolism, signaling, and effector function, and integrates posttranscriptional regulation not captured by mRNA measurements. Cross‑species proteomic comparisons further provide a powerful means to identify conserved functional programs and to define species‑specific divergences that influence the translational relevance of murine pDC models.

Indeed, differences in pDC signaling and interferon regulation between mice and humans have been reported. Murine pDCs exhibit strong dependence on IFNAR‑mediated positive feedback to amplify TLR9‑driven interferon responses, whereas human pDCs display more context‑dependent deployment of TLR7 and TLR9 signaling, with both amplifying and regulatory interferon feedback mechanisms described [3, 4]. How such functional distinctions relate to underlying proteomic and metabolic organization remains poorly understood.

Emerging work has highlighted cellular metabolism, nutrient availability, and metal‑ion homeostasis as critical regulators of immune cell state and function [5, 6]. Iron, in particular, is an essential cofactor for mitochondrial respiration, redox reactions, and multiple enzyme systems, yet excess unbound iron poses a risk of oxidative stress and ferroptotic cell death [7]. Cellular iron uptake is mediated mainly through the transferrin receptor (TFRC), which binds transferrin‑loaded iron, while ferroportin (SLC40A1) is the only known mammalian iron exporter [8]. Beyond iron acquisition, TFRC has been implicated in endocytic trafficking and viral entry in several immune contexts [9].

In this study, motivated by the observation that pDCs express unusually high levels of TFRC, we investigated pDC metabolism with a focus on iron handling using deep, absolute quantitative proteomics of primary murine pDCs isolated ex vivo. We aligned the murine pDC proteome to equivalent human pDC datasets and to proteomes of conventional dendritic cell subsets to define conserved and divergent metabolic features. This multilayered analysis demonstrates that pDCs across species exhibit constitutively high TFRC expression and robust transferrin‑iron uptake, yet do not accumulate increased total or stored cellular iron. The detection of ferroportin within the pDC proteome suggests that iron export counterbalances sustained iron import, establishing a dynamic iron flux rather than an iron storage state. Together, these findings provide new insight into the metabolic organization of pDCs and raise important questions regarding noncanonical roles of iron trafficking in innate immune function.

## 2 Results

### 2.1 pDC Express High Levels of the Transferrin Receptor

**FIGURE 1:**
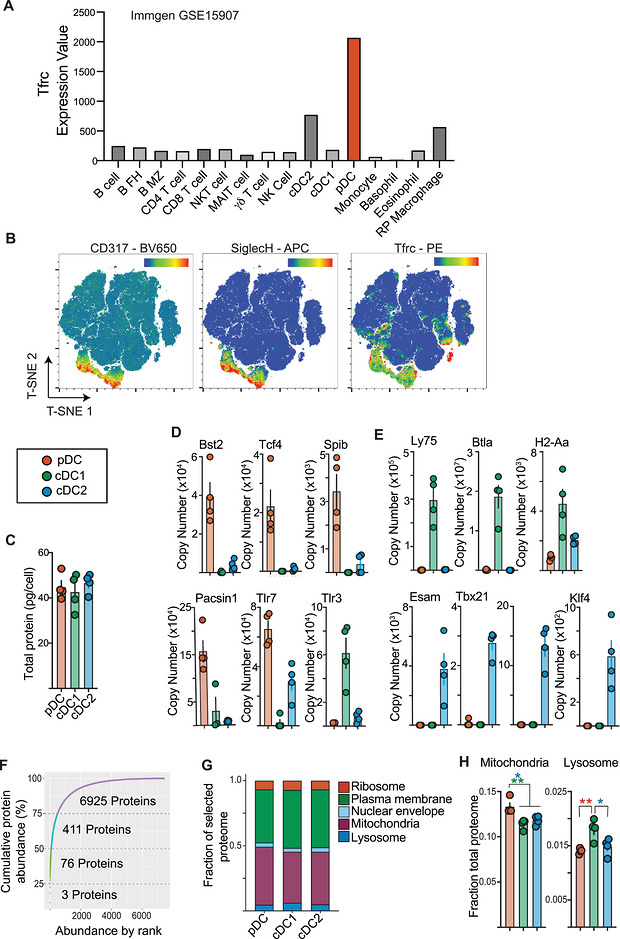
Quantitative proteomic profiling of murine pDCs. **(A)** Tfrc mRNA expression in the indicated immune cell subsets under steady‐state conditions, analyzed using the ImmGen microarray dataset (GSE15907). **(B)** Flow cytometry analysis of splenic immune cells from B16‐Flt3L‐treated C57BL/6 mice. t‐SNE dimensionality reduction was applied to singlet, live/dead‐negative splenocytes, revealing a distinct population of CD317^hi^SIGLEC‐H^hi^Tfrc^hi^ cells. Panels show expression of CD317 (left), SIGLEC‐H (middle), and Tfrc (right). (**C–G**) Proteomic analysis was performed on FACS‐sorted splenic pDC, cDC1, and cDC2 isolated from naïve C57BL/6 mice. Total protein per cell calculated from proteomic data (C). Absolute protein copy numbers per cell for (D) pDC and (E) cDC surface markers and lineage‐defining transcription factors. Proteins ranked by abundance (*x*‐axis) plotted against cumulative abundance (*y*‐axis) with quartile thresholds indicated (F). Cumulative bar chart showing distribution of total protein abundance by cellular compartment, based on Gene Ontology terms: plasma membrane (GO:0005886), nuclear envelope (GO:0005635), mitochondria (GO:0005739), ribosome (GO:0005840), lysosome (GO:0005764) (G). Quantification of the mitochondrial and lysosomal proteome in pDC and cDC, using GO terms of each protein to assign a compartment (H). Data are representative (B), mean (A), or mean + SEM of 3 or 4 (C–H) separate biological replicates. Data are analyzed using a one‐way ANOVA with Tukey posttest (H) (*p < 0.05; **p < 0.01; ***p < 0.001).

Murine plasmacytoid dendritic cells (pDC) are the subset with the highest expression of Tfrc mRNA, when comparing immune subsets in the basal state using the Immgen GSE15907 dataset (Figure 1A). This raised the question of whether iron uptake is important for the immune functions of pDC. To confirm that elevated Tfrc mRNA is translated into elevated TFRC proteins, we stained splenocytes with antibodies against cell lineage markers and the TFRC and generated a t‐SNE plot. In these experiments, DC subsets were expanded in vivo following the injection of Flt3‐releasing B16 cells because DC cells are very low frequency in the spleen (Figure S1A). The t‐SNE plot shows very strong staining for TFRC that colocalizes with the pDC, CD317^high^, and SiglecH^high^ cells (Figure 1B). Based on these interesting observations, we wanted to explore the metabolic machinery of pDC in a robust and nonbiased way using quantitative proteomics. To avoid any artifacts that might arise due to the in vivo expansion protocol, DC subsets were sorted from the splenocytes of naïve mice using an ultra‐low input proteomic pipeline as used in collaboration with the University of Dundee.

### 2.2 Establishing the Steady‑State Proteome of Murine pDCs Directly Ex Vivo

pDCs were identified by flow cytometry as lineage negative (CD3, CD19), CD11c^int^, CD317^+^, and Siglec H^+^ cells, and 10,000 pDCs were sorted ex vivo directly from splenocytes by FACS for proteomic analysis (Figure S1B). Absolute protein copy numbers per cell were estimated using the histone ruler method [10]. Proteomes of cDC1 and cDC2 dendritic cell subsets generated, in parallel with pDC, were used here as comparative dendritic cell references.

Across four pDC biological replicates, a total of 7352 proteins were identified, of which 5430 proteins were consistently detected in all replicates (Figure S1D). Quantification accuracy was assessed by enumerating the number of unique and razor peptides used for protein identification. Proteins were classified as highly accurate when quantified with more than five peptides, moderately accurate with two to five peptides, and low accuracy when supported by fewer than two peptides. Using these criteria, the majority of detected proteins were quantified with high confidence, with approximately 61% classified as high accuracy, 16% as medium accuracy, and 23% as low accuracy.

The total protein content of murine pDCs was estimated, from the proteome data, at approximately 45 pg per cell, with minimal variation across replicates (Figure 1C). This value is comparable to the total protein content of cDC1 and cDC2 subsets quantified in parallel (Figure S1C). Consistent with this, pairwise comparisons of protein copy numbers between replicates demonstrated extremely high reproducibility, with Pearson correlation coefficients exceeding 0.99 (Figure S1E; data shown for replicate 1 vs. replicate 2). Overall, approximately 84% of proteins were detected in at least three out of four biological replicates, underscoring the robustness of the dataset (Figure S1D).

To confirm the cellular specificity of the proteomic datasets, expression of lineage‑defining markers was examined across pDC, cDC1, and cDC2 proteomes. The pDC proteome was highly enriched for canonical pDC transcription factors Tcf4 (E2‐2) and Spib [11]; surface markers including Bst2 (CD317), Tlr7, and Pacsin1 [11, 12] (Figure 1D). Also confirming the purity of the pDC proteomic was the absence of certain proteins, including Tlr3 and lineage markers for cDC1 (Ly75, Btla, and H2‑Aa) and cDC2 markers (Esam, Klf4, and Tbx21) (Figure 1E). Collectively, these analyses demonstrate that the proteomic data accurately reflect the steady‑state proteome of purified murine pDCs.

To explore the organizational features of the pDC proteome, proteins were ranked by mean abundance, and their cumulative contribution to total protein copies was calculated. Despite the identification of thousands of proteins, proteome composition was highly concentrated, with 79 proteins accounting for more than 50% of total cellular protein abundance (Figure 1F). This pattern is consistent with the inherently uneven distribution of protein abundance in cells, where a relatively small number of high‐abundance proteins dominate the proteome, as reported for multiple immune cell types, including T cells [13]. The most abundant proteins were involved in core cellular functions, including chromatin organization (core histones), cytoskeletal structure (actin), protein synthesis and folding (Rps27a and Ppia), and redox regulation (Prdx1) (Table S1). This is as expected for the proteome of immune cells and similar to other datasets on NK cells and described for CD8 T cells previously.

Finally, proteomic allocation across major subcellular compartments was assessed using gene ontology annotations. Proteins associated with the plasma membrane, nuclear envelope, mitochondria, ribosome, and lysosome were quantified and compared across dendritic cell subsets (Figure 1G; Table S2). This analysis revealed that mitochondrial proteins represented a larger proportion of the pDC proteome compared with cDC1 and cDC2, whereas cDC1 cells exhibited a relative expansion of the lysosomal proteome (Figure 1H). These findings provide insight into the distinct organizational and metabolic features underpinning dendritic cell subset identity.

### 2.3 Strong Correlation Between Proteome of Human and Murine pDC

**FIGURE 2:**
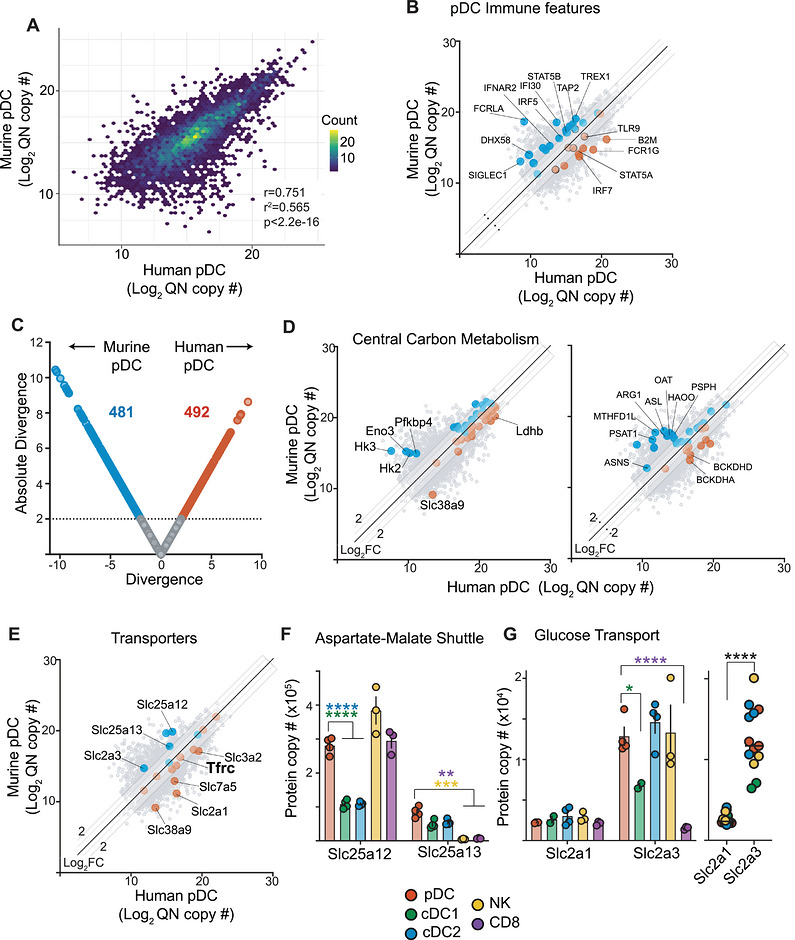
Comparative analysis of murine and human pDCs. **(A–E)** Orthologs in the murine pDC proteome and human pDC proteome (PXD004352) were identified, data were quartile‐normalized and aligned to give 4485 matched proteins. Log_2_ normalized abundance for murine (*y*‐axis) and human (*x*‐axis) proteins was plotted, and correlation analysis was performed (A). Species‐specific divergent proteins were overlaid on an *x*, *y* plot in categories for pDC immune features and are seen as those outwit the 2× Log_2_ fold change lines shown. (B), central carbon metabolism (D), and transporters (E). A volcano plot was prepared of murine and human pDC proteomes for protein divergence versus absolute divergences, showing (C). (F, G) Protein copy numbers of murine transporters associated with the aspartate‐malate shuttle (F) and glucose import (G, left), comparing DC subsets, naïve CD8 T cells, and NK cells. Overall comparison of Slc2a1 and Slc2a3 copy numbers in splenic cells, with subsets color‐coded (G, right). Data are mean (A–E) and mean ± SEM of 4 separate biological replicates (F, G). Data are analyzed using a Pearson's correlation analysis (A), a two‐way ANOVA with a Dunnett's posttest (F, G left) or a student's *t*‐test (G, right). (**p < 0.01; ***p < 0.001; ****p < 0.0001).

A landmark study by Guilliams et al. [14] demonstrated that dendritic cell (DC) subsets are conserved across tissues and species using combined flow and mass cytometry approaches. To assess the evolutionary conservation of plasmacytoid dendritic cells (pDCs), we compared the murine pDC proteome generated here with a previously published human pDC proteomic dataset deposited in the ProteomeXchange Consortium (PXD004352). Human protein identifiers were converted to murine orthologs and aligned with the murine dataset. Protein copy numbers were normalized, orthologous proteins were identified, and the datasets were merged, yielding a matrix of 4485 matched proteins with four biological replicates per species. Correlation analysis revealed a strong positive relationship between murine and human pDC proteomes (Pearson *r * =  0.751; Figure 2A). The calculated *R* ^2^ value of 0.56 indicates that approximately 56% of proteome‐wide variance is shared between species. Notably, this analysis was restricted to proteins with annotated orthologues between human and mouse, which are expected to be evolutionarily conserved, and thus likely contributes to the observed level of concordance. This correlation was highly significant (*p* < 2.2 × 10^−16^), indicating that the observed similarity is unlikely to occur by chance.

### 2.4 Expression of PAMPs and DAMPs in Human and Murine pDCs

Quantitative proteomic comparison of human and murine plasmacytoid dendritic cells (pDCs) revealed selective, pathway‑specific differences rather than global proteome shifts. Murine pDCs showed higher relative abundance of proteins involved in intracellular antigen processing and nucleic‑acid handling, including FCRLA, IFI30, TREX1, TAP2, DHX58, and SIGLEC1, with several exceeding 3× log_2_ differences (Figure 2B).

In contrast, key pDC lineage and differentiation markers (TCF4, SPIB, IRF8, BST2, and CD40) exhibited minimal interspecies variation. Murine cells also displayed increased abundance of interferon signaling components such as IFNAR2, STAT5B, and IRF5, alongside murine‑only detection of IFNAR1 and NOD1, consistent with tonic type I interferon signaling described in mouse pDCs [15, 16]. Conversely, human pDCs were enriched for proteins associated with endosomal nucleic‑acid sensing and interferon production, including IRF7, TLR9, FCER1G, STAT5A, and B2M (Figure 2B). Additional pattern‑recognition and regulatory proteins (e.g., TLR1/4/5/6, CLEC4E, CLEC7A, DDX41, IFI16, MB21D1, TMEM173, and SIGLEC5/6) were detected only in the human dataset. This profile aligns with evidence that human pDC interferon production critically depends on endosomal TLR7/9 signaling [3, 17]. Notably, CD74 abundance was similar between species, indicating conservation of core MHC‑II machinery despite divergence in associated accessory proteins. Overall, these data support conserved pDC identity across species with distinct biases in antigen processing, interferon signaling, and pattern‑recognition pathways.

### 2.5 Divergence in Metabolic Pathways Between Human and Murine pDC

To further interrogate species‑specific differences, mean protein abundances were quartile‑normalized to a common reference distribution to remove global abundance biases while preserving relative protein rankings. Following log_2_ transformation, per‑protein divergence was calculated as the difference between human and murine abundance, with proteins displaying absolute divergence values greater than 2 (that is >fourfold difference) defined as species‑enriched. Applying the divergence criteria identified 481 murine‑enriched and 492 human‑enriched proteins (Figure 2C; Table S3). Pathway enrichment analysis of species‑enriched proteins revealed significant overrepresentation of metabolic pathways in both datasets (Figure S2A,B). To explore these differences further, proteins involved in central carbon metabolism were mapped onto the murine‑to‑human proteome comparison. Most metabolic enzymes were highly expressed and broadly conserved; however, murine pDCs showed increased expression of several glycolytic proteins, including hexokinase 2, hexokinase 3, enolase, and Pfkfbp4 (Figure 2D, left). As Pfkfbp4 regulates intracellular fructose‑2,6‑bisphosphate levels and potently activates phosphofructokinase‑1, its increased abundance in murine pDC suggests a readiness for rapid glycolysis upon immune activation. Human pDCs showed a particular enrichment for Slc38a9 (>eightfold difference), a lysosomal arginine and glutamine sensor that regulates mTORC1 activity, consistent with known metabolic control mechanisms in human pDC function [18].

To further characterize metabolic differences between species, we extended the analysis to additional amino‑acid metabolic pathways, first comparing murine and human pDCs. Quantitative analysis of amino‑acid metabolic proteins revealed coordinated but pathway‑specific differences between species. Murine pDCs showed higher relative abundance of enzymes involved in serine‐glycine and one‑carbon metabolism (MTHFD1L, PSAT1, and PSPH), arginine and ornithine metabolism (ARG1, ASL, and OAT), and histidine and aromatic amino‑acid catabolism (HAL, HAAO, and KYNU), consistent with enhanced representation of amino‑acid interconversion pathways (Figure 2D, right). In contrast, human pDCs exhibited a higher abundance of enzymes associated with branched‑chain amino acid degradation (BCKDHA and BCKDHB). Together, these data indicate that murine pDCs are characterized by broader amino‑acid remodeling capacity.

The transport of nutrients represents key steps in cellular metabolism. The TFRC was highly expressed in both murine and human pDC (Figure 2E). Murine pDCs were enriched for the mitochondrial aspartate‐glutamate transporters SLC25A12 and SLC25A13, core components of the aspartate‐malate shuttle (Figure 2E). The expression of these transporters was assessed in other murine splenic subsets. Expression of Slc25a12 was higher in murine pDCs than in cDC1 and cDC2 subsets (Figure 2F). In contrast, human pDCs were enriched for both subunits of the large neutral amino‑acid transporter LAT1 (SLC7A5 and SLC3A2), consistent with an increased capacity for import of extracellular amino acids (Figure 2E). Notably, SLC7A5 is closely linked to mTORC1 signaling, which has previously been shown to regulate key aspects of human pDC function [18]. In addition, murine and human pDCs displayed differential expression of glucose transporter isoforms, with human pDCs expressing higher levels of SLC2A1 (GLUT1) and murine pDCs expressing higher levels of SLC2A3 (GLUT3). Within murine splenocytes, pDC expressed higher levels of SLC2A3 compared with cDC1 and naïve CD8 T cells (Figure 2G, left). All subsets measured had low expression of SLC2A1. Overall, SLC2A3 was consistently expressed at higher levels than SLC2A1 for the innate immune cells measured; that is, DC subsets and Natural Killer cells (Figure 2G).

### 2.6 pDCs are Enriched in Proteins for Iron Storage and Fe–S Cluster Assembly

**FIGURE 3:**
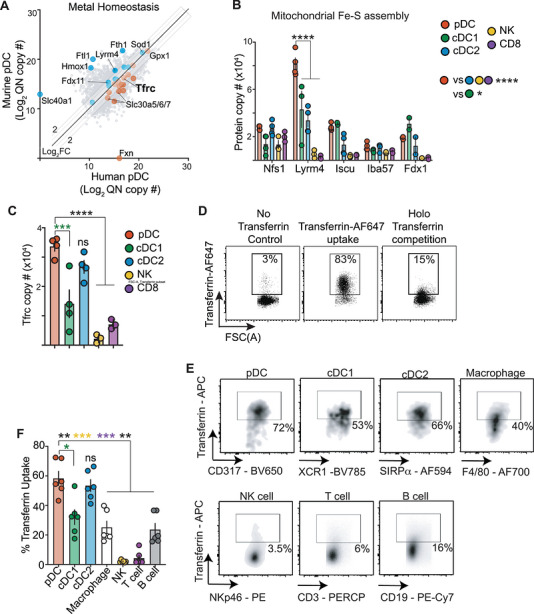
Machinery for iron storage and Fe–S assembly elevated in murine pDC. **(A)** An *x*, *y* graph of murine (*y*‐axis) and human (*x*‐axis) protein orthologs, plotting log_2_ quartile‐normalized protein copy numbers. Overlaid are proteins associated with metal homeostasis. Species divergent proteins are those that outwit the 2× Log_2_ fold change line shown. (**B, C**) Protein copy numbers for the protein components of the mitochondrial Fe–S assembly complex (B), and the transferring receptor (TFRC) (C), comparing DC subsets and naïve CD8 T cells and NK cells. (**D–F**) Transferrin‐iron uptake analysis using an AlexaFluor‐647 tagged transferrin probe. Specificity of uptake is proven using holo‐transferrin competition control (D). Comparison of the frequency of transferrin uptake positive splenic cells comparing CD317^+^ pDC, XCR1^+^ cDC1, SIRPα cDC2, F4/80 macrophages, NKp46^+^ NK cells, CD3^+^ T cells, and CD19^+^ B cells (E, F). Data are mean (A), mean ± SEM (B, C, F), or representative (D, E) of four independent experiments. Data are analyzed using a one‐way ANOVA with Tukey's post hoc test (C, F) and a two‐way ANOVA with a Dunnett's posttest (*p < 0.05; **p < 0.01, ***p < 0.001; ****p < 0.0001).

The pDC proteome was enriched for mitochondrial proteins compared with cDC subsets (Figure 1H). Because metal ions serve as essential cofactors for numerous mitochondrial proteins, including components of the electron transport chain, we next considered proteins involved in metal‑ion homeostasis. Murine pDCs showed higher abundance of both ferritin subunits, which store iron atoms in intracellular protein cages (Figure 3A). Murine pDCs were also enriched for components of the iron–sulfur (Fe–S) cluster assembly machinery, most notably LYRM4, which participates in the formation of Fe–S clusters required for the activity of multiple enzyme classes. In contrast, human pDCs exhibited enrichment of zinc transporter proteins, including SLC30A5, SLC30A6, and SLC30A7 (Figure 3A). pDCs showed significantly higher overall abundance of the Fe–S assembly machinery at the pathway level. However, when assessed at the level of individual proteins, only LYRM4 reached statistical significance (Figure 3B).

pDC expressed higher TFRC protein than cDC1, NK cells and CD8 T cells but had similar overall protein copies to cDC2 (Figure 3C; Figure S3). This was investigated further using a transferrin uptake assay that uses a transferrin protein tagged with a fluorophore. This approach is accurate because the mechanism of uptake in receptor‐mediated endocytosis accommodates the fluorophore tag. Specificity was confirmed using a competition control of excess holo‐transferrin (Figure 3D). pDC showed the highest frequency of transferrin uptake compared with other splenic subsets. This was statistically compared with cDC1, macrophages, T cells, NK cells, and B cells (Figure 3E,F). The only subset that showed comparable uptake was cDC2, consistent with similar TFRC protein expression (Figure 3D,F).

### 2.7 pDC Do Not Have Increased Iron Storage, or Total Iron Molecules Per Cell

**FIGURE 4:**
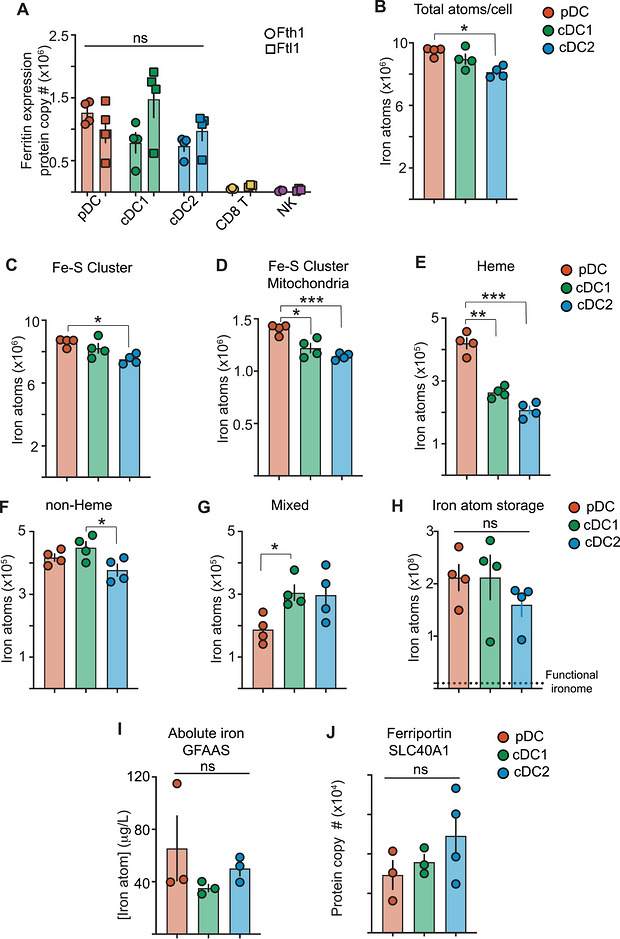
pDC does not accumulate iron atoms. **(A)** Protein copy numbers for ferritin heavy (Fth1) and light (Ftl1) subunits comparing splenic DC subsets and naïve CD8 T cells and NK cells. (**B–H**) Proteomic analysis of iron‐binding proteins calculated the number of iron atoms per cell (B). Similarly, iron atoms in total proteins (C) and mitochondrial proteins (D) containing Fe–S clusters were calculated. Iron in heme (E), nonheme (F), and mixed (G) contexts was also calculated. Iron atom storage in ferritin nanocages (H) was calculated. (I) Absolute iron atom content for sorted splenic DC subsets was measured using GFAAS. (J) Protein copy numbers for ferroportin (SLC40A1) in splenic DC subsets. Data are mean ± SEM of 3–4 individual experiments. Data are analyzed using a one‐way Brown–Forsythe and Welch ANOVA and Dunnett's T3 posttest (D, E, G, I, J), a Kruskal–Wallis test and Dunn's posttest (B, C, H), a one‐way ANOVA with Tukey posttest (F), and a two‐way ANOVA and Dunnett's posttest (A). (*p < 0.05; **p < 0.01; ***p < 0.01; ns, nonsignificant).

pDCs do not exhibit increased iron storage or total cellular iron content relative to other dendritic cell subsets. Iron's capacity to reversibly donate and accept electrons underpins its central role in redox catalysis and oxygen binding; however, excess unbound iron promotes reactive oxygen species generation via the Fenton reaction. Consequently, intracellular iron is tightly regulated through coordination by iron‑binding proteins, sequestration within ferritin complexes, or export via the iron transporter ferroportin. To investigate why pDCs exhibit constitutive transferrin‑mediated iron uptake, we examined intracellular iron fate across dendritic cell subsets. Cross‑species comparison revealed that murine pDCs were enriched for ferritin proteins (Figure 3A). However, comparison of murine splenic immune subsets demonstrated that ferritin protein abundance was similar across all DC subsets and markedly higher than in lymphocytes (Figure 4A).

To quantify functional iron utilization, proteomic abundances were mapped to a comprehensive murine iron‑binding protein catalogue and scaled using published protein‑specific iron stoichiometries to generate a quantitative, gene‑level cellular ironome. Iron contributions were then aggregated by binding modality (Fe–S clusters, heme, and nonheme iron) to compare iron allocation across dendritic cell subsets [19]. Of 186 iron‑binding proteins detected in the proteomic dataset, 166 were present in pDCs. Collectively, these proteins define the functional ironome of each cell type. The total iron atoms associated with functional iron‑binding proteins were significantly higher in pDCs compared with cDC2s, but not cDC1s (Figure 4B).

This increase in the functional ironome was primarily due to Fe–S containing proteins, which accounted for the largest proportion of iron atoms associated with proteins (Figure 4C). Fe–S proteins were enriched with mitochondrial proteins, which is consistent with the elevated mitochondrial proteome observed for pDC (Figure 1). Indeed, when analysis was restricted to mitochondrial Fe–S proteins, pDCs showed significantly higher iron allocation than both cDC1 and cDC2 subsets (Figure 4D). Though heme‐bound proteins accounted for a much smaller proportion of protein‐bound iron, pDC were also significantly enriched in this category relative to both cDC1 and cDC2 (Figure 4E). Elevated heme iron content reflected selective upregulation of cytochrome‑ and NADPH oxidase‐associated proteins, particularly CYBB, CYBA, and CYB561 family members, rather than uniform changes across the heme proteome. cDC1s displayed a modest increase in nonheme iron, driven by cumulative elevations in mono‑iron regulatory enzymes, including epigenetic dioxygenases and lipid‑modifying enzymes, rather than mitochondrial or redox‑associated iron systems (Figure 4F). Proteins containing both Fe–S and heme cofactors (mixed iron proteins), including ABCB7, ETFDH, and CISD family members, were selectively reduced in pDCs (Figure 4G).

Iron storage capacity was assessed by estimating ferritin nanocage numbers from ferritin heavy and light chain copy numbers. Assuming a conservative 50% iron loading of ferritin complexes, the calculated number of ferritin‑stored iron atoms per cell was comparable across all dendritic cell subsets (Figure 4H). Notably, ferritin‑stored iron was present in substantial excess relative to iron associated with functional iron‑binding proteins (dotted line in Figure 4H), indicating that differences in iron handling between DC subsets reflect differential allocation to functional iron systems rather than differences in total iron storage. Consistent with the 10‐fold greater iron storage over iron in the functional ironome and the equivalent storage capacity of DC subsets, the absolute cellular iron atom content quantified by graphite furnace atomic absorption spectroscopy (GFAAS) was comparable across pDC, cDC1, and cDC2 populations (Figure 4I). Together, these data demonstrate that overall iron abundance is similar between DC subsets and that ferritin storage capacity is conserved, while subset‑specific differences arise from redistribution of iron into distinct functional iron‑dependent protein systems.

To resolve the apparent paradox of elevated transferrin‑mediated iron uptake in pDCs despite equivalent total cellular iron content compared with cDCs, we examined expression of the iron exporter ferroportin (SLC40A1), the only known mammalian protein capable of mediating cellular iron efflux. Proteomic analysis revealed that Slc40a1 was expressed by pDCs, cDC1s, and cDC2s, but was undetectable in other splenic populations such as NK cells or CD8 T cells (Figure 4J). Together, these data indicate that pDCs sustain high rates of transferrin‑iron uptake without increasing intracellular iron burden, consistent with concurrent iron export via Slc40a1.

### 2.8 Iron Availability Is Not Essential for pDC IFNα Production

**FIGURE 5:**
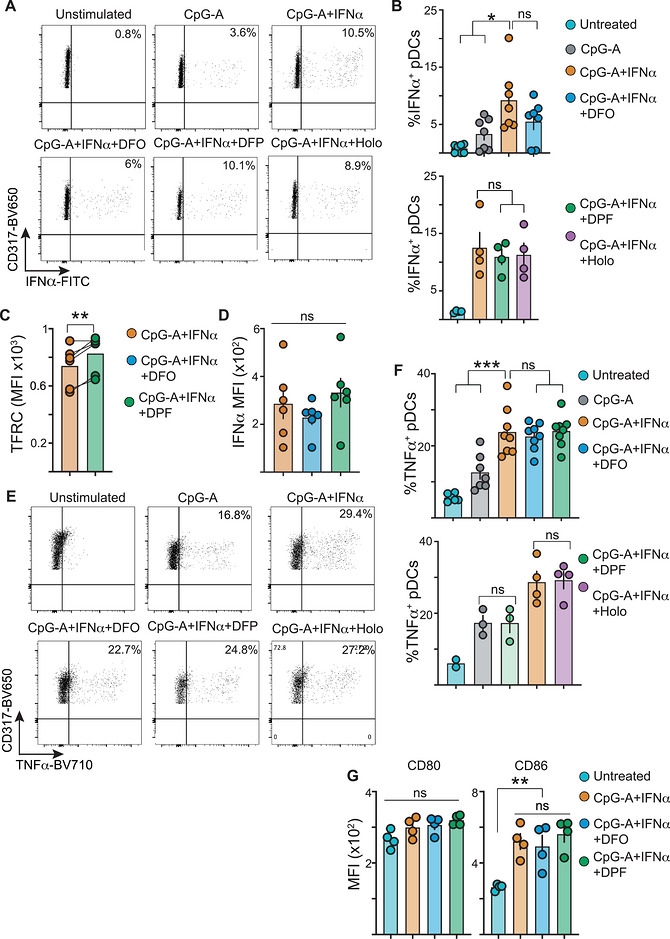
Iron chelation does not impair pDC IFNα production. **(A–F)** Flow cytometry analysis of splenic pDC from naïve C57BL/6 mice with and without stimulation with CpG‐A (4 µg/mL) alone or with IFNα (1 µg/mL) for 6 h in the presence or absence of iron chelators deferoxamine (DFO; 20 µM, A–F) or deferiprone (DFP; 10 µM, A–F) or holo‐transferrin (500 µg/mL, A, B, F). Extracellular CD80 and CD86 (G) or intracellular IFNα and TNFα expression were measured. Data are representative (A, E), shown as paired samples (C) or mean ± SEM (B, D, F) of 3–7 biological replicates. Data are analyzed using an RM one‐way ANOVA with Tukey posttest (B, D, F) or a paired students *t*‐test (C) (*p < 0.05; **p < 0.01; ***p < 0.001; ns, nonsignificant).

Limiting iron availability has been reported to impair effector functions in T cells, NK cells, and macrophages [20, 21, 22]. Given the constitutive transferrin‑mediated iron uptake observed in pDCs, we tested whether iron availability influences Type I interferon production following TLR9 stimulation. CD317^+^SiglecH^+^ pDCs produced IFNα in response to CpG‑A stimulation of splenocytes, consistent with TLR9 activation (Figure 5A). In response to CpG‑A alone, the frequency of IFNα‑positive pDCs was low, in agreement with previous reports that Type I IFN responses are initiated by a limited number of pDCs and subsequently amplified via IFNAR1 signaling [23]. Indeed, co‑stimulation with CpG‑A and IFNα significantly increased the number of IFNα‑producing pDCs (Figure 5A).

To assess the role of iron availability in pDC cytokine production, splenocytes were stimulated with CpG‑A and IFNα in the presence or absence of the iron chelators deferoxamine (DFO) or deferiprone (DFP), and IFNα production was measured by flow cytometry (Figure 5B). Neither DFO nor DFP reduced the frequency of IFNα‑producing pDCs. Importantly, DFP treatment increased expression of TFRC on pDCs, confirming effective intracellular iron deprivation (Figure 5C). Conversely, supplementation with excess holo‑transferrin did not enhance IFNα production. IFNα production on a per‑cell basis, measured as mean fluorescence intensity in IFNα^+^ pDCs, was likewise unchanged following iron chelation or iron supplementation (Figure 5D). In parallel experiments, TNF production was also unaffected by DFO, DFP, or holo‑transferrin treatment following CpG‑A and IFNα stimulation (Figure 5E,F). Collectively, these data demonstrate that iron availability does not regulate pDC pro‑inflammatory cytokine production.

### 2.9 pDCs Maintain High Surface Expression of TFRC

**FIGURE 6:**
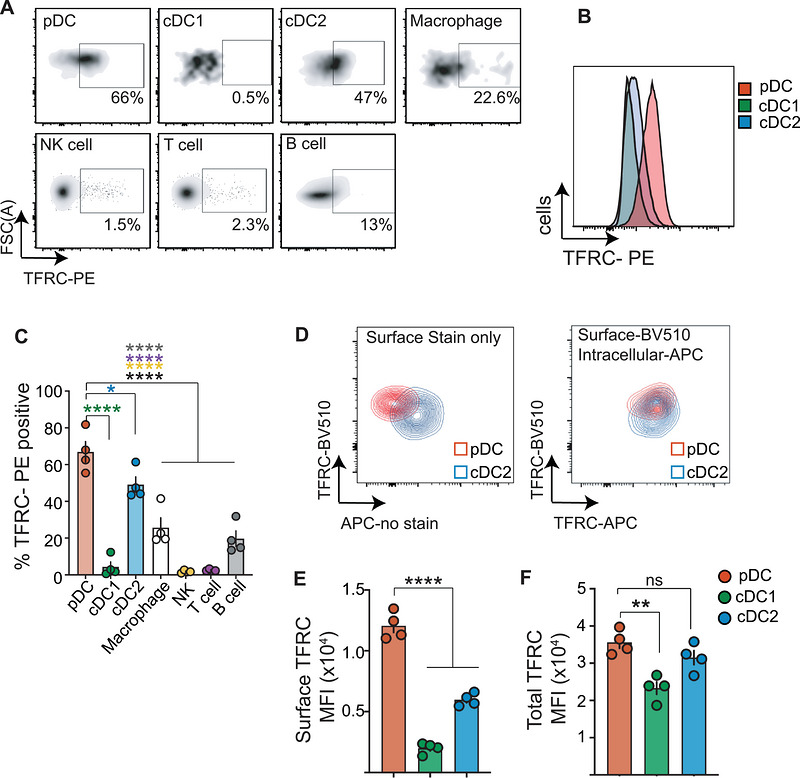
pDC maintains elevated surface TFRC expression. **(A–D)** Splenocytes were stained for immune subsets and TFRC and analyzed by flow cytometry. Representative density plots are shown (A), a histogram overlay of pDC, cDC1, and cDC2 subsets (B), and pooled data (C). To visualize surface TFRC and intracellular TFRC pools, pDC were stained with TFRC‐BV510, fixed and permeabilized, and stained with a different antibody clone, TFRC‐APC (D, right). Control cells were not stained after permeabilization (D, left). Overlaid are pDC1 (red) and cDC2 (blue). (E) Pooled data of surface staining and total TFRC staining (surface plus intracellular), cell‐specific FMO background subtracted. (F, G) Data are representative (A, B, D) and mean ± SEM of four biological replicates (C, F, G). Data are analyzed using one‐way ANOVA with a Dunnett's posttest (F, G). (*p < 0.05; **p < 0.01; ****p < 0.0001; ns, nonsignificant).

Transferrin uptake assays assess the maximal capacity for iron import and require short‑term culture in serum‑free conditions, which promote redistribution of TFRC to the cell surface. However, iron uptake under steady‑state conditions is likely to depend on basal surface TFRC expression. pDCs expressed significantly higher levels of surface TFRC than all other splenic populations analyzed, with cDC2s showing the next highest expression (Figure 6A–C). When total TFRC protein was assessed by combining surface and intracellular staining, pDCs and cDC2s displayed comparable overall TFRC abundance, consistent with proteomic measurements (Figure 6D,E). Thus, pDCs preferentially maintain a large proportion of their TFRC at the cell surface without exhibiting increased iron loading or iron‑binding capacity, suggesting that surface TFRC expression in pDCs is uncoupled from cellular iron accumulation and may serve functions beyond iron acquisition.

## 3 Discussion

This study presents a comprehensive ex vivo proteomic analysis of murine splenic plasmacytoid dendritic cells (pDCs), deliberately avoiding potential artifacts associated with in vivo expansion, in vitro differentiation, or prolonged culture. Given increasing evidence that immune cells differentiated or maintained in vitro undergo substantial metabolic and proteomic remodeling compared with their in vivo counterparts [24, 25, 26], this ex vivo approach strengthens the physiological relevance of our findings and provides an accurate baseline for understanding pDC biology in the steady state.

Using the histone ruler method [10], we achieved absolute protein quantification, enabling global analysis of pDC proteome structure and allocation. As observed in other immune cell types, proteome composition was highly skewed, with a small fraction of highly abundant proteins accounting for more than half of total cellular protein mass [27]. These dominant proteins were predominantly associated with core cellular functions, including chromatin organization, cytoskeletal structure, and intermediary metabolism. Absolute quantification further enabled subcellular compartment analysis, revealing that the pDC protein mass allocated to mitochondria is significantly greater than that in cDC1 and cDC2. Meanwhile, cDC1 had a larger proportion of proteins associated with the lysosome. Previous functional studies have suggested that pDC activation relies on distinct metabolic programs compared with conventional dendritic cells, including differential engagement of glycolysis and mitochondrial metabolism. Our proteomic data now provide a structural basis for these observations by revealing disproportionate allocation of protein mass to mitochondrial pathways in the steady state [28].

Cross‐species proteomic comparison revealed a high degree of conservation between murine splenic and human circulating pDCs (Pearson's *r* = 0.75), in line with prior transcriptional and cytometric analyses establishing conserved dendritic cell lineages across species [14, 29]. Although the compared cells derive from different anatomical compartments, murine pDCs are known to recirculate between blood and lymphoid organs, mitigating concerns regarding tissue‐specific bias. At the same time, our data highlight meaningful species‐specific differences. Human pDCs exhibited relative enrichment for LDHB, enabling lactate metabolism through respiration, and Slc38a9, an amino acid sensor for the control of mTORC1 activity. Murine pDCs showed greater enrichment of proteins associated with glycolysis, including Hk2 and 3, Eno2, and Pfkbp4, an enzyme that primes pDC for glycolytic flux upon activation.

As immune cell activation states are increasingly recognized to be shaped by nutrient availability and transporter expression [30], we examined nutrient transport systems across species. Several transporters, including the transferrin receptor (TFRC), were conserved between human and murine pDCs, whereas others, such as the large neutral amino acid transporter SLC7A5 and the glucose transporter SLC2A1, were enriched in human pDCs. Murine pDC favored the expression of the SLC2A3 glucose transporter. Enrichment of SLC7A5 is consistent with its established role in mTORC1 activation and IFN‑I production, potentially under IL‑3‑dependent regulation [17]. While murine pDCs have less amino acid transporter expression, they were enriched for proteins involved in amino acid interconversion, providing the ability to synthesize different amino acids within the cell.

Because iron uptake is typically associated with proliferative or metabolically active immune cells [21, 31, 32, 33], the high expression of TFRC in nondividing pDCs prompted further investigation of intracellular iron utilization. Normalized dendritic cell proteomics were filtered to iron‐binding proteins, annotated by iron class, corrected for stoichiometric iron content per protein, and summed to quantify class‐specific and total iron loads across pDC, cDC1, and cDC2 samples. Data presented for Fe–S, heme, and nonheme iron‐containing proteins revealed distinct ironome patterns between DC subsets. Overall, the functional ironome of pDCs was significantly greater than that of cDC2, but not cDC1. This difference was due to increased heme and Fe–S containing proteins, especially those associated with the mitochondria, consistent with an overall larger mitochondrial proteome in pDC. However, the capacity for iron storage in ferritin nanocages was similar in all DC subsets; each DC had the capacity to store 10‐fold more iron atoms than iron atoms associated with proteins.

Consistent with these proteomic predictions, direct quantification of iron atoms per cell revealed no difference between pDCs and conventional DC subsets, despite the latter lacking elevated TFRC expression or constitutive transferrin uptake. Although ferroportin expression in DCs was substantially lower than in professional systemic iron‑handling cells such as enterocytes and red pulp macrophages [9], its presence in pDCs suggests that sustained iron export may counterbalance constitutive transferrin‑iron uptake, resulting in net iron homeostasis rather than accumulation.

These findings raise the possibility that high TFRC expression in pDCs may reflect functions beyond meeting intracellular iron demand. Several viruses, including influenza virus, SARS‑CoV‑2, and New World arenaviruses, are known to exploit TFRC for cellular entry [9, 34, 35], and TFRC has also been implicated in IgA1‑mediated endocytosis [36]. Moreover, TFRC traffics through early endosomal compartments, where TLR7 and TLR9 signaling is initiated. Enhanced transferrin receptor cycling could, in principle, influence endosomal dynamics relevant to nucleic acid sensing, potentially biasing signaling toward interferon‑producing pathways rather than maturation programs [37, 38]. This provides a rationale for why pDC retain a much larger proportion of TFRC at the cell surface. While these possibilities remain speculative, they offer a conceptual framework linking elevated surface TFRC expression with the unique antiviral sensing behavior of pDCs [37].

To directly test whether iron availability itself influences pDC effector function, we assessed cytokine production under conditions of iron chelation. Neither deferoxamine nor deferiprone impacted IFNα or TNFα production in response to CpG‑A plus IFNα stimulation, indicating that acute iron deprivation does not constrain pDC cytokine output. This contrasts with proliferative lymphocytes, where TFRC upregulation is tightly coupled to iron demand during cell division [13, 21], and further supports the notion that TFRC abundance in mature pDCs reflects a distinct functional context. Therefore, our data do not support a direct role for iron availability in regulating pDC cytokine production under the conditions tested, nor do they establish a causal role for TFRC in antiviral sensing. Rather, they redefine how iron handling is organized in pDCs at baseline.

A limitation of this study is that proteomic abundance does not directly report enzyme activity, metabolite flux, or subcellular localization dynamics. While absolute proteomics enables quantification of iron‑binding capacity and protein allocation, resolving how iron flux is dynamically regulated during pDC activation will require complementary metabolic and imaging approaches.

In summary, this study identifies conserved, high TFRC expression in murine and human pDCs that is uncoupled from intracellular iron accumulation, iron storage capacity, or cytokine production. Instead, pDCs appear to maintain dynamic iron flux through coordinated iron uptake and export, without net accumulation. These findings redefine how iron handling is organized in pDCs and suggest that TFRC abundance may reflect specialization of endocytic or trafficking pathways rather than elevated metabolic iron demand. Further experimental work will be required to determine whether TFRC contributes directly to antiviral sensing or antigen handling in pDCs and to establish how iron dynamics shape innate immune responsiveness.

## 4 Materials and Methods

### 4.1 Mice

C57BL/6J were purchased from Harlan (Bicester, UK) or Charles River or were bred in‐house. All mice used for this project were between 8 and 12 weeks of age, male or female. Experiments were conducted in compliance with the Finlay lab project license, with ethical approval from the Trinity College Dublin University ethics committee and the Animal Research Ethics Committee from the Health Products Regulatory Authority (HPRA). Animals were culled by CO_2_ inhalation or cervical dislocation. Organs were harvested in the comparative medicine unit (CMU), and carcasses were disposed of in accordance with the guidelines described by the TCD CMU.

### 4.2 In Vivo DC Expansion

To expand the dendritic cell compartment in vivo, B16‐Flt3l secreting melanoma cells were grown on one flank following subcutaneous injection of 2.5 × 10^6^ B16‐Flt3l cells in 100 µL PBS (B16‐Flt3l cells were a gift from Bart Everts lab, Leiden University). After 10–12 days of tumor growth, mice were euthanized, the spleen harvested, and processed as described below.

### 4.3 DC Isolation and Culture

Spleens were mechanically disrupted before the addition of 50 µL of collagenase D (Roche, #11088866001) (final concentration 1 mg/mL) and DNase I (Sigma, #D4263) (final concentration 2000 U/mL) for 30 min at 37°C, 5% CO_2_. Then, the cells were filtered through 70 µm sterile filters and centrifuged. The resulting pellet was resuspended in ACK (150 mM sodium chloride, 10 mM potassium bicarbonate, 100 µM ethylenediaminetetraacetic acid; Sigma) for 5 min to lyse red blood cells. RPMI (Sigma, #R5886) was added to neutralize the ACK buffer. Following centrifugation, the splenocytes were resuspended in RPMI supplemented with 2 mM L‐Glutamine (Sigma, #G8540), 10% heat‐inactivated fetal calf serum, 50 µM β‐mercaptoethanol (Gibco, #31350), 50 IU/mL penicillin, and 50 IU/mL streptomycin: (complete RPMI). For splenocyte experiments, 1 × 10^6^ splenocytes/mL were seeded in 96‐well U‐bottom cell culture plates in 200 µL of complete RPMI. Where indicated, splenocytes were treated with CpG‐A (ODN‐1585, Invitrogen, #tlrl‐1585) (4 µg/mL), CpG‐B (ODN‐1826, Invitrogen, #tlrl‐1826) (4 µg/mL), and deferoxamine (Sigma, #D9533) (20 µg/mL).

### 4.4 Flow Staining

For analysis of surface markers, cells were first incubated with FC block (Biolegend, #101320) for 5 min in FACS buffer (PBS (Gibco, #10010023) + 0.25% BSA (Sigma, #A9467) + 0.1% Sodium azide (Sigma, #71289) and stained for 20 min at room temperature in the dark. For intracellular Tnfα and IFNα detection in pDCs, cells were stimulated with 4 µg/mL CpG‐A. After 2 h of stimulation, exocytosis was blocked using Golgi Plug (BD Biosciences, #555029, contains Brefeldin A) to capture intracellular cytokines. Cells were stained with surface markers before fixation and permeabilization using Cytofix/Cytoperm (BD Biosciences, #554714) for 20 min in the dark at 4°C. Intracellular antibody staining was performed in 1× Perm Wash (BD Biosciences, #554723) for 30 min at room temperature in the dark. Samples were acquired on LSR Fortessa and analyzed using FlowJo software (TreeStar, version 10).

### 4.5 Flow Cytometry Antibodies

CD11b‐BV605 (Biolegend, #135517, Clone: AFS98) CD11c‐BV421 (BD Biosciences, #562782, Clone: HL3), CD19‐BV650 (BD Biosciences, #563235, Clone: 1D3), CD19‐PE‐Cy7 (BD Biosciences, #552854, Clone: 1D3) CD3‐PerCP (Miltenyi, #130‐120‐826, Clone: REA641), CD317‐BV650 (BD Biosciences, #747605, Clone: 927), TFRC‐PE (BD Biosciences, #553267, Clone: C2), TFRC‐BV510 (Clone C2), TFRC‐APC (Clone R17217), F4/80‐AF700 (Biorad, #MCA497A7, Clone: BM8.1), Siglec‐H‐FITC (Biolegend, #129603, Clone: 551), TNFα‐PE‐Cy7 (Invitrogen, #25‐7321‐82, Clone: MP6‐XT22), IFNα‐AF488 (Biotechne, #FAB2451G, Clone: 1056928), NKp46‐PE (EBiosciences, #12‐3351‐82, Clone: 29A1.4), SIRP‐α—AF594 (Biolegend, #144020, Clone: P84), XCR1‐BV785 (Biolegend, #148225, Clone: ZET), LIVE/DEAD Aqua (Invitrogen, #L34957)

### 4.6 Transferrin Uptake

Transferrin uptake was determined by measuring the uptake of Alexa fluor‐conjugated transferrin (Invitrogen, #T23366) by flow cytometry. Cells were washed in serum‐free RPMI supplemented with 0.5% BSA. Subsequently, cells were incubated in RPMI + 5% BSA for 1 h at 37°C, 5%. After incubation, cells were incubated with or without 5 µg/mL Alexa Fluor‐conjugated Transferrin for 10 min at 37°C, 5% CO_2_. As controls, cells were also incubated with Holo‐transferrin (500 µg/mL) (Sigma, #T4132), or uptake was performed on ice. Uptake was ceased by washing the cells with ice‐cold acid wash (PBS supplemented with 150 mM NaCl and 20 mM citric acid, pH 5) followed by washing in ice‐cold RPMI + 0.5% BSA. Cells were stained with surface antibodies as described above, and samples were acquired live on the BD LSRII Fortessa and analyzed using FlowJo software (TreeStar, version 10).

### 4.7 Iron Quantification

Total cellular iron in dendritic cells was quantified by graphite furnace atomic absorption spectroscopy (GFAAS) on a PinAAcle 900Z spectrometer (PerkinElmer). Lysed cells were digested in equal volumes of 50% nitric acid at 70°C for 2 h, cooled, and centrifuged at 6000×*g* for 5 min. Iron levels were measured by atomic absorption at 248.33 nm, with each sample analyzed in technical triplicate. Mean iron concentrations (µg/L) were calculated by extrapolation from a standard curve prepared with known concentrations of iron.

### 4.8 Processing Low Cell Numbers for Proteomic Analysis

To enable mass‑spectrometric analysis of low cell numbers, cells were sorted under stringent conditions to minimize protein contamination. Cells were harvested and stained in RPMI supplemented with 0.5% fetal calf serum and sorted with a stringent forward/side‑scatter threshold into low‑bind tubes pre‑loaded with freshly prepared lysis buffer. cDC1 were identified as LIVE/DEAD^−^, CD3^−^, CD19^−^, CD11c^+^, MHCII^hi^, XCR1^+^, SIRPα^−^ cells, cDC2s were identified as LIVE/DEAD^−^, CD3^−^, CD19^−^, CD11c^+^, MHCII^hi^, XCR1^−^, SIRPα^+^, and pDCs were identified as LIVE/DEAD^−^, CD3^−^, CD19^−^, CD11c^int^, and CD317^+^ SiglecH^+^ cells. Cells (10,000 pDC and cDC2 and 2000 cDC1) were sorted into lysis buffer (RIPA buffer, 50 mM TEAB, 5 mM TCEP, 0.5% benzonase), snap‐frozen, and sent to Dundee for further processing. Proteomics sample preparation was performed by Howden, Sinclair, and Cantrell Labs, and mass spectrometry was run with the Proteomics Facility at the University of Dundee, UK. Briefly, peptides from frozen cell lysates were prepared using a modified SP3 method [39].

### 4.9 LC‐MS Analysis

Peptides were analyzed using an Astral Orbitrap Mass Spectrometer (Thermo Fisher), coupled with a Vanquish nano‐LC (Thermo Scientific). LC buffers were the following: buffer A and weak buffer wash (0.1% formic acid in Milli‐Q water (v/v)), buffer B and strong buffer wash (80% acetonitrile, 0.1% formic acid in Milli‐Q water (v/v)). Samples (equivalent of 200 ng) were loaded onto a trap column (Pep Map Neo C18, 5 µm, 300 µm × 5 mm, Thermo Scientific) equilibrated in buffer A with the following loading settings mode: combine‐control, flow 200 μ/min, pressure 800 Bar, and loading volume 50 µL. Peptides were separated on a C18 column (Aurora Ultimate, 25 cm × 75 µm ID, 1.7 µm particle size) maintained at 50°C. The column was equilibrated at 1% buffer B. Peptides were eluted at a variable flow rate, starting at 0.5 µL/min and decreasing to 0.4 µL/min. The gradient was as follows: from 1% to 8% buffer B in 0.2 min; from 8% to 35% buffer B over 30 min; and from 35% to 45% buffer B over 6 min. The flow rate was then increased from 0.4 to 0.5 µL/min over 0.4 min while buffer B was increased from 45% to 99%.

The data were acquired using an easy spray source operated in positive mode with spray voltage at 2.100 kV, and the ion transfer tube temperature at 280°C. The MS was operated in DIA mode. A scan cycle comprised a full MS scan (m/z range from 380 to 980), with Orbitrap resolution at 2400000, RF lens at 40%, AGC target set to custom, normalized AGC target at 500%, absolute AGC value set to 5.00e6, maximum injection time 3 ms, and microscan set to 1. MS survey scan acquired in profile mode was followed by MS/MS DIA scan events using the following parameters: DIA window type set to auto, isolation window 2, window overlap set to 0, window placement optimization on, number of scan events 300, collision energy type normalized, HCD collision energy 25%, detector type Astral, scan range 150–2000, normalized AGC target 500%, absolute AGC target 5.000e4, maximum injection time 3 ms, microscan set to 1, loop control time 0.6 s and DIA data were collected in centroid mode.

### 4.10 Processing of Proteomic Data

The data were processed, searched, and quantified using Spectronaut 19 using the directDIA option. The direct DIA data were searched against the UniProt mouse database (SwissProt and Trembl). Protein copy number quantification was performed in the Perseus software package [40]. Mean copy numbers were estimated using the “proteomic ruler” described in the study by Wiśniewski et al. [10]. The accuracy of quantification was established using the following guidelines: proteins categorized as high accuracy had more than eight unique and razor peptides and a ratio for unique/unique + razor of ≥0.75; proteins categorized as medium accuracy had at least three unique and razor peptides, and a ratio for unique/unique + razor of ≥0.5; and any proteins below these thresholds were classified as low accuracy.

Quantitative proteomic data from pDC, cDC1, and cDC2 subsets were used to estimate relative subcellular composition. Following normalization and aggregation of protein abundances to the gene level, proteins were assigned to major subcellular compartments based on curated UniProt and Gene Ontology Cellular Component annotations. Each protein was assigned a dominant localization to avoid overrepresentation of multi‑localized proteins. For each sample, compartmental protein abundances were summed and normalized to total cellular protein abundance to calculate proportional subcellular composition. Mean compartmental profiles were calculated across replicates to enable comparison between dendritic cell subsets.

### 4.11 Cross‑Species Proteomic Normalization and Comparison

Protein copy‑number estimates from human primary pDCs (four steady‑state biological replicates) were extracted and normalized using a median‑ratio size‑factor approach analogous to DESeq2. For each protein, a geometric mean across replicates was calculated after excluding zero values. Replicate‑specific size factors were derived as the median ratio of observed copy number to the geometric mean and used to scale each replicate, correcting for global differences in protein loading.

Human‐mouse orthologous gene pairs were defined using the NCBI HomoloGene database, restricting analyses to unambiguous one‑to‑one orthologs. Human and mouse proteomic datasets were filtered to retain only detected orthologous proteins. Where multiple protein entries mapped to the same gene, abundances were collapsed by averaging replicate values to generate a single gene‑level estimate.

Mean copy numbers were calculated per gene and per species, and values were log_2_‑transformed following the addition of a pseudocount. Cross‑species similarity was assessed using Pearson correlation analysis and visualized using scatter plots, hexbin density plots, and correlation matrices across all replicates.

To assess proteome divergence independently of scale, human and mouse gene‑level abundances were additionally subjected to quantile normalization. Log_2_ fold‑change between species was calculated for each ortholog, and proteins were categorized as human‑enriched, mouse‑enriched, or conserved using a fourfold threshold. Quantile‑normalized divergence metrics were visualized using volcano plots, and ranked protein lists were exported for downstream analysis.

To identify biological pathways underlying cross‑species differences in pDC proteomes, gene‑level divergence was calculated following quantile normalization of human and mouse orthologous protein abundances. Proteins were classified as human‑ or mouse‑enriched using a log_2_ divergence threshold of ≥2 or ≤−2, respectively. Human‑enriched genes were analyzed directly, whereas mouse‑enriched proteins were first mapped to their human orthologs using one‑to‑one assignments from the NCBI HomoloGene database. Gene Ontology Biological Process and KEGG pathway enrichment analyses were performed separately for each gene set using clusterProfiler, with Benjamini–Hochberg correction (adjusted *p* < 0.05). KEGG pathway gene identifiers were converted back to human gene symbols for interpretation.

### 4.12 Quantitative Iron Proteome Analysis

Normalized proteomic abundance data from murine pDC, cDC1, and cDC2 subsets (four biological replicates per subset) were intersected with a curated murine iron‑binding protein catalogue from [19]. Iron‑binding proteins were classified by cofactor type (Fe–S cluster, heme, nonheme iron, and mixed iron) using published iron‑interaction annotations. To account for sporadic missing values attributed to mass‑spectrometric dropout, missing abundances within each DC subset were imputed using the mean value across replicates of the same subset. Protein abundances were collapsed to a single gene‑level value by summation, and gene‑level abundances were scaled by published protein‑specific iron stoichiometries to calculate iron atoms associated with each protein per sample. Iron contributions were aggregated by binding modality and summed to derive subset‑specific functional ironomes. Ferritin‑mediated iron storage was estimated independently by summing ferritin heavy (Fth1) and light (Ftl1) chain abundances to infer ferritin nanocage numbers, assuming 24 subunits per complex and 50% maximal iron loading (2250 iron atoms per cage).

### 4.13 Statistical Analysis

Details of the statistical analyses performed can be found in the figure legends. Data are expressed as mean ± standard error of the mean (SEM) or standard deviation (SD) unless stated otherwise. *p*‐values were calculated using a two‐tailed Student's *t*‐test for two‐group comparisons. A one‐sample *t*‐test was used to compare more than two groups against one sample. To compare multiple samples, one‐way ANOVA with Dunnett's, Tukey's, or Sidak's posttest or two‐way ANOVA with Dunnett's posttest was used as indicated.

## Author Contributions

C.C., S.O'S., and D.F. conceived and designed the study. C.C., S.O'S., C.L., S.W., and S.K. performed experiments and acquired data. L.S. contributed to proteomics methodology and data acquisition. C.C., S.O'S., C.L., S.W., S.K., and D.F. analyzed and interpreted the data. D.F. and L.S. analyzed the proteomic dataset. D.F. and S.C. supervised the study. S.O'S., C.C., and D.F. wrote the manuscript with input from all authors. All authors reviewed and approved the final manuscript.

## Funding

This work was supported by the Taighde Éireann/Research Ireland (22/FFP‐A/10326 and IRCLA/2023/1402) and European Research Council (ERC‐CoG‐770769). C.C. was supported by a Government of Ireland studentship (GOIPG/2022/81).

## Conflicts of Interest

The authors declare no conflicts of interest.

## Supporting information




**Supporting File 1**: eji70219‐sup‐0001‐tableS1.xlsx.

**Supporting File 2**: eji70219‐sup‐0002‐tableS2.xlsx.

**Supporting File 3**: eji70219‐sup‐0003‐tableS3.xlsx.

**Supporting File 4**: eji70219‐sup‐0004‐figuresS1‐S3.pdf.

## Data Availability

The quantitative human proteomics data used in this study were previously deposited in the ProteomeXchange Consortium repository under accession number **PXD004352**. The mass spectrometry proteomics data have been deposited to the [PRIDE/ProteomeXchange Consortium] with the dataset identifier [PXD079623]. The proteomic data can also be browsed at ImmPres.co.uk. Additional datasets supporting the findings of this study, including NK and CD8 proteomes, are available from the corresponding author upon reasonable request.
