# Supplementary material for: Plasmacytoid Dendritic Cells Exhibit High Transferrin Receptor Expression Without Iron Accumulation
Source: Eur J Immunol. 2026 Jun 17;56(6):e70219. doi: 10.1002/eji.70219 (PMC13273928; doi:10.1002/eji.70219)
Supplement: Supplementary file 4 — Supporting File 4: eji70219‐sup‐0004‐figuresS1‐S3.pdf. [file EJI-56-e70219-s001.pdf]

## Supplementary Figure 1

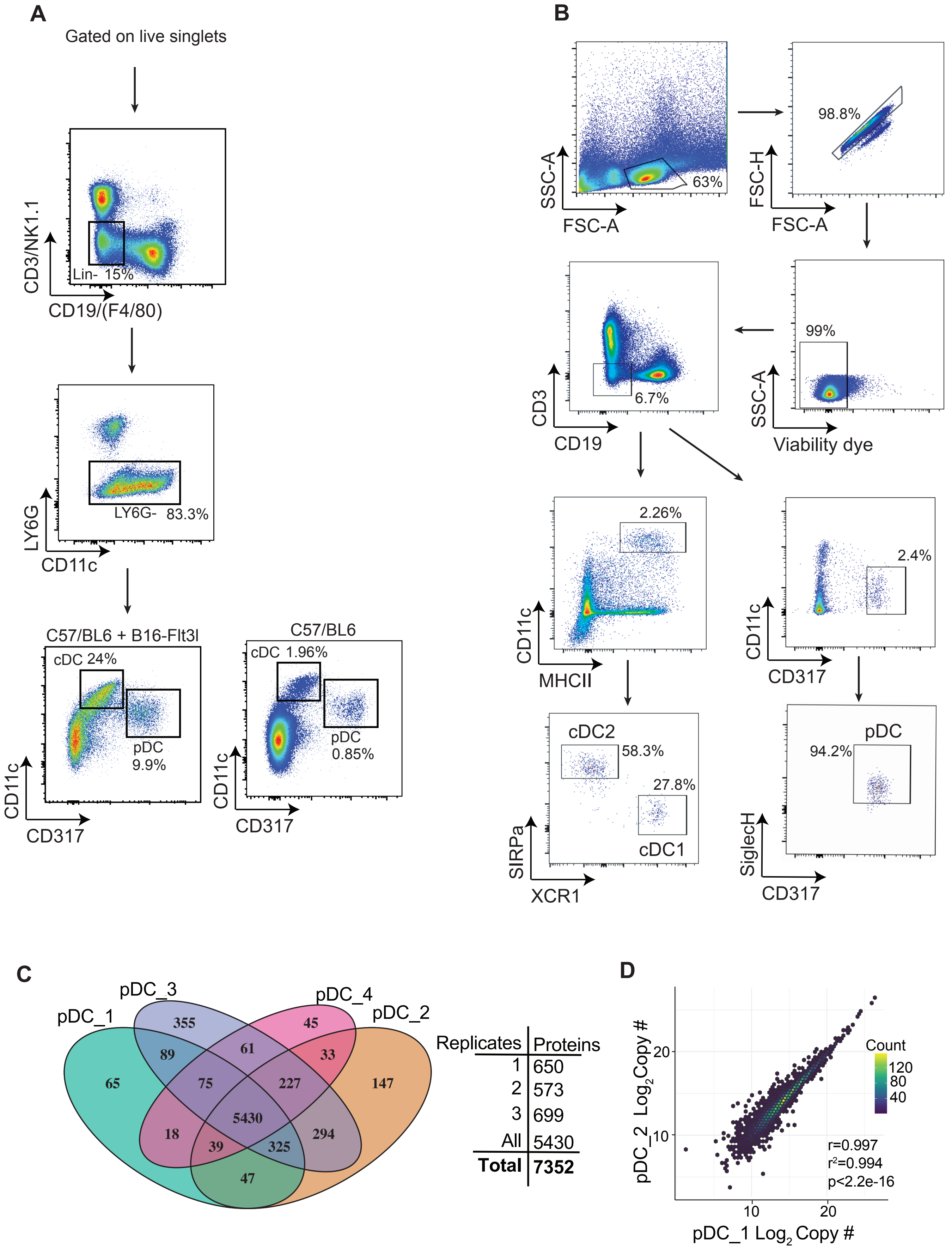

**Supplementary Figure 1: Characterization of splenic pDC.**
(A) Representative flow plot for splenic pDC with and without in vivo expansion due to the s.c injection of B16-Flt3 cells. (B) Flow cytometry gating strategy for pDC, cDC1 and cDC2 from splenocytes from naïve C57BL/6 mice (C,D) Proteomic analysis was performed on FACS-sorted splenic pDC, cDC1 and cDC2 isolated from naïve C57BL/6 mice. (C) Venn diagram showing shared and unique proteins identified across pDC biological replicates. (D) Correlation plot of Log2 protein copy number between biological replicates 1 and 2. Data is representative of proteomics pairwise comparisons and analysed using a Pearson correlation.

**Supplementary Figure 2**

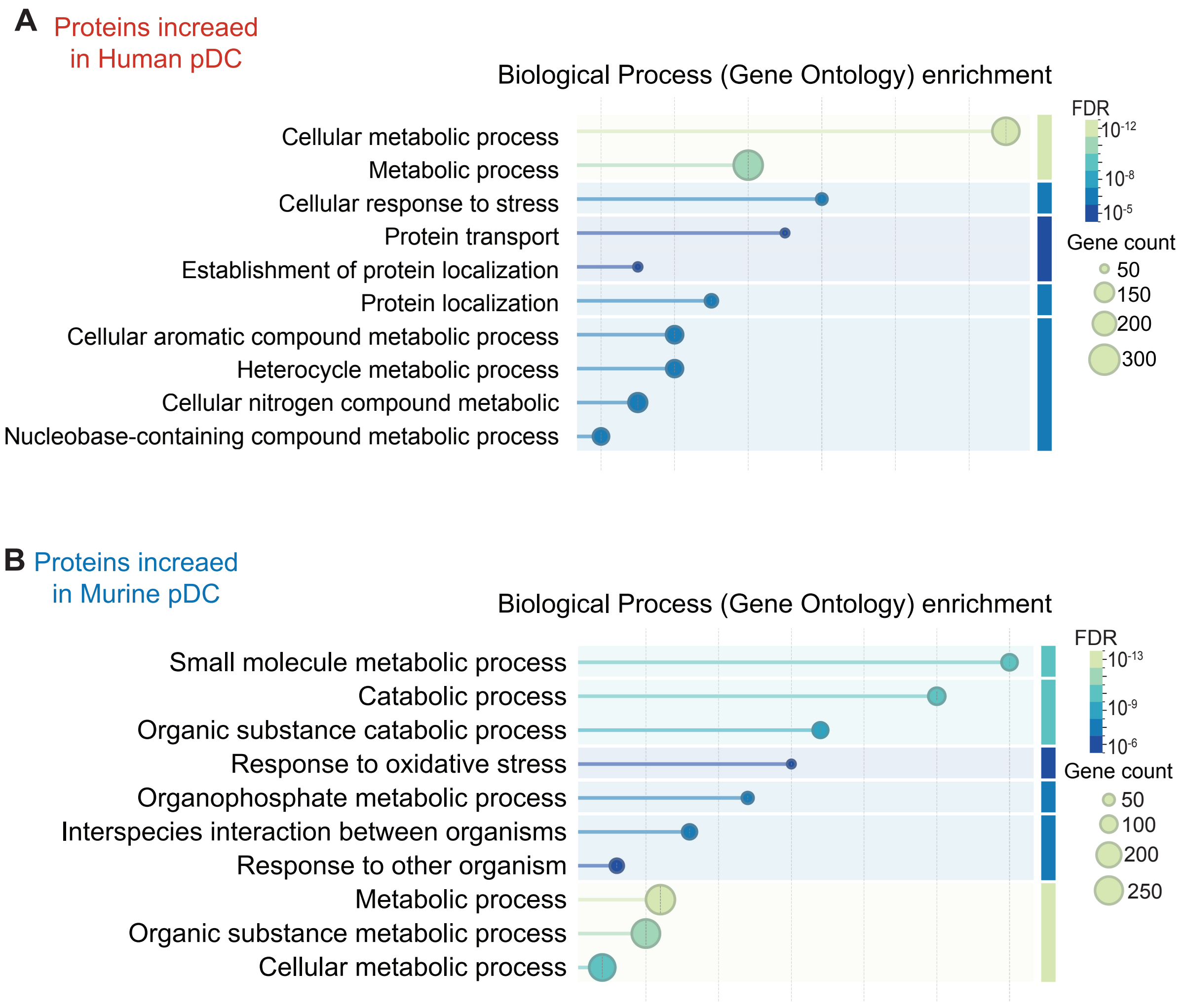


**Supplementary Figure 2: Pathway analysis of divergent proteins in murine and human pDC.** (A,B) Orthologues in murine pDC proteome and human pDC proteome (PXD004352) were identified, data was quartile normalized and aligned to give 4,485 matched proteins. Log2 normalized abundance for murine (y-axis) and human (x-axis) proteins were plotted and correlation analysis performed. Log2 normalized abundance for murine and human proteins were plotted and correlation analysis performed. Species-specific divergent proteins were identified as those greater than 2x Log2 fold change. Species divergent proteins were interrogated using GO pathway analysis. Significantly enriched pathways for Human enriched proteins (A) and murine enriched proteins (B) are shown.

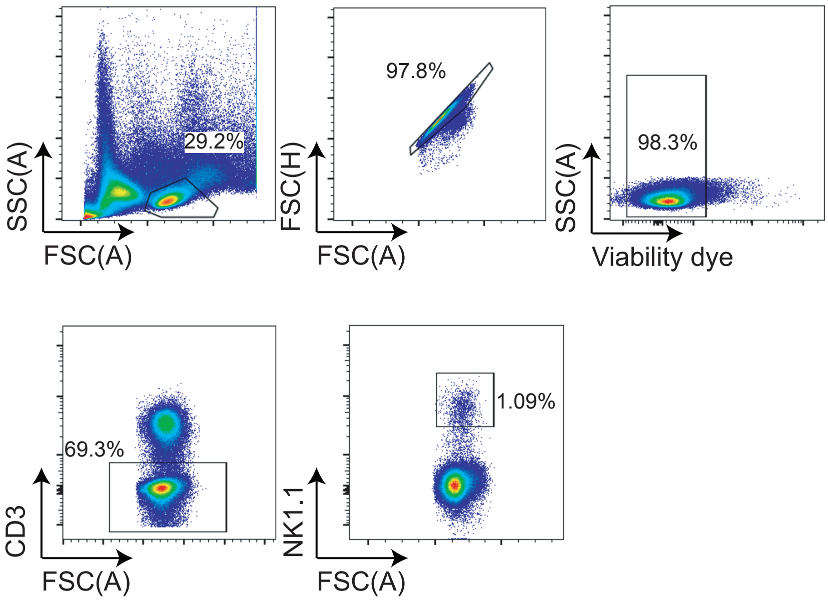


**Supplementary Figure 3: Gating strategy for NK cells proteomics**
Flow cytometry gating strategy for NK cells from splenocytes of naïve C57BL/6 mice
